# Cardiac Rehabilitation for Aboriginal and Torres Strait Islander people in Western Australia

**DOI:** 10.1186/s12872-016-0330-3

**Published:** 2016-07-13

**Authors:** Sandra Hamilton, Belynda Mills, Shelley McRae, Sandra Thompson

**Affiliations:** Western Australian Centre for Rural Health, University of Western Australia, 35 Stirling Highway, Crawley, WA 6009 Australia; National Heart Foundation of Australia, 334 Rokeby Road, Subiaco, WA 6009 Australia

**Keywords:** Indigenous, Cardiac Rehabilitation, Secondary Prevention, Western Australia, Access, Health Pathways, Alternative Methods

## Abstract

**Background:**

Cardiovascular disease (CVD) is a leading cause of morbidity and mortality in Australia. Australian Aboriginal and Torres Strait Islander (Indigenous) people have higher levels of CVD compared with non-Indigenous people. Cardiac Rehabilitation (CR) is an evidence-based intervention that can assist with reducing subsequent cardiovascular events and rehospitalisation. Unfortunately, attendance rates at traditional CR programs, both globally and in Australia, are estimated to be as low as 10-30 % and Indigenous people are known to be particularly under-represented. An in-depth assessment was undertaken to investigate the provision of CR and secondary preveniton services in Western Australia (WA) with a focus on rural, remote and Indigenous populations. This paper reports on the findings for Indigenous people.

**Methods:**

Cardiac rehabilitation and Aboriginal Medical Services (n = 38) were identified for interview through the Heart Foundation *Directory of Western Australian Cardiac Rehabilitation and Secondary Prevention Services 2012.* Semi-structured interviews with CR coordinators were conducted and included questions specific to Indigenous people.

**Results:**

Interviews with coordinators from 34 CR services (10 rural, 12 remote, 12 metropolitan) were conducted. Identification of Indigenous status was reported by 65 % of coordinators; referral and attendance rates of Indigenous patients differed greatly across WA. Efforts to meet the cultural needs of Indigenous patients varied and included case management (32 %), specific educational materials (35 %), use of a buddy or mentoring system (27 %), and access to an Aboriginal Health Worker (71 %). Staff cultural awareness training was available for 97 % and CR guidelines were utilised by 77 % of services.

**Conclusion:**

The under-representation of Indigenous Australians participating in CR, as reported in the literature and more specifically in this study, mandates a concerted effort to improve services to better meet the needs of Indigenous patients with CVD as part of closing the gap in life expectancy. Improving access to culturally appropriate CR and secondary prevention in WA must be an important component of this effort given the high rates of premature cardiovascular disease affecting Indigenous people. Our findings also highlight the importance of good systematic data collection across services. Health pathways that ensure continuity of care and alternative methods of CR delivery with dedicated resources are needed.

## Background

Cardiovascular disease (CVD) is a leading cause of morbidity and mortality and the leading disease category for health-care expenditure in Australia [1, 2]. Cardiovascular disease accounted for 31 % of all deaths in 2011 with coronary heart disease (CHD) accounting for 15 % of all deaths and 47 % of CVD deaths, many of which are premature and preventable [3]. Australian Aboriginal and Torres Strait Islander (Indigenous) people have higher levels of CVD compared with non-Indigenous people. In 2011-13, Indigenous Australians were 1.6 times more likely to report CVD and their rate of acute coronary events was 2.5 times higher than non-Indigenous Australians [4, 5]. Cardiovascular disease also occurs at a younger age in Indigenous Australians, some 10 years earlier than in non-Indigenous people, with rates in 35-54 year age group four times greater than non-Indigenous Australians [6]. These discrepancies in rates are profound and have been reported for Indigenous Western Australians for myocardial infarction,[7] atrial fibrillation,[8] and heart failure [9].

Cardiac Rehabilitation (CR) and secondary prevention is well-recognised as effective management for cardiac patients and results in improved clinical and behavioural outcomes including reductions in subsequent cardiovascular events and hospitalizations and improved survival [10–12]. However, more than 40 % of CHD events, 50 % of CHD death and 35 % of nonfatal myocardial infarctions occur in people with existing CHD [13]. Unfortunately participation rates at traditional CR programs, both globally and in Australia, are estimated to be as low as 10-30 % with an under-representation of Indigenous people [10, 14–18]. Vulnerable patient populations such as Indigenous patients, women, older patients, socioeconomic disadvantaged, minority ethnic populations, rural patients and those with more prevalent comorbidities are less likely to adhere and complete CR [17, 19, 20]. Low participation rates represent healthcare service (health professional and provision/systems of care) failures and patient-related barriers to attendance, participation or adherence despite their being referred [15–31]. Failure to refer patients to CR is a major barrier to participation [32] with referral rates varying from 10 – 60 % [25, 32–35]. Factors positively related to referral have been identified and include younger age, male, Caucasian, patients undergoing percutaneous intervention or coronary artery bypass grafting and a positive attitude to CR by the referring physician or health professional [25, 32–36]. Barriers to CR use for Indigenous Australians have been identified and include: extended family responsibilities [37, 38]; issues with family support and understanding [38]; sociocultural appropriateness of the program [37]; lack of knowledge about CR [37, 38]; low income [37]; connection between colonialism and health services [37]; disempowering health messages in the media [37]; and the younger age of the Indigenous patients [37].

Approximately, 13 % of all Indigenous people live in Western Australia (WA), comprising approximately 3.1 % of the total WA population [39]. Rural and remote regions of WA have a substantially higher Indigenous population, especially the Kimberley (40.0 %), Pilbara (12.0 %) and Midwest regions (10.0 %) [39]. In a 2008 study, Thompson et al. examined and described health professionals’ awareness, implementation, and perspectives on the barriers to the implementation of the 2005 National Health and Research Council’s (NHMRC) *Strengthening Cardiac Rehabilitation and Secondary Prevention for Aboriginal and Torres Strait Islander peoples: A Guide for Health Professionals* [40, 41]. The guide’s key measures for successful CR in Indigenous Australians are summarised in Table 1 [40–42]. Twenty-four health professionals from 17 services in WA were interviewed; suboptimal awareness and limited uptake of the NHMRC guidelines was identified [40]. Inpatient and outpatient CR services were limited for Indigenous patients and use of Indigenous resources was low. Workforce issues, cultural competence and service linkages emerged as factors impacting on the design and delivery of CR services for Indigenous people in WA. It was concluded that that the disproportionate burden of CVD mortality and morbidity in Indigenous people required urgent attention, dedicated resources and alternative approaches to CR [40].

**Table 1:** Key measures for successful cardiac rehabilitation in Indigenous Australians [40–42]

1. Ensure cultural safety is integral to the core business of an organisation and supported at all levels within the organisation.
2. Involve an Aboriginal Health Worker or Liaison Officer and family members in the care of Indigenous Australians and develop flexible approaches in a setting that is comfortable to them, highlighting the importance of CR.
3. Draw on existing CR and secondary prevention services as appropriate and engage with the local community networks.
4. Ensure community involvement and control in planning, implementing and evaluating programs, including the development of culturally appropriate educational resources.
5. Develop and sustain partnerships between organisations, including a referral network.
6. Take the specific needs of Indigenous Australians into consideration in planning and delivering CR and secondary prevention and develop supportive protocols, policies and procedures that address these needs.
7. Develop specialist educational resources and training for continuing professional development and support of all health professionals in heart care, including Aboriginal Health Workers, Liaison Officers and Allied Health Assistants.

The recently released Australian Cardiovascular Health and Rehabilitation (ACRA) *Core Components of Cardiovascular Disease Secondary Prevention and Cardiac Rehabilitation 2014* [43] and the Department of Health, Western Australia *Cardiovascular Rehabilitation and Secondary Prevention Pathway Principles for Western Australia* [44] acknowledge the disparities in the management and secondary prevention of CVD for Indigenous Australians.

This in-depth assessment investigated the provision of CR and secondary prevention services in WA with a focus on rural, remote and Indigenous populations. This paper reports on the findings for Indigenous services, in particular service provision, culturally appropriate approaches for Indigenous patients including availability of Indigenous staff, staff cultural awareness training, and CR guideline use.

## Methods

### Study design

A mixed methods study utilising structured interviews of CR Coordinators was undertaken. Quantitative and qualitative interview questions were based on the *National Heart Foundation of Australia and the Australian Cardiac Rehabilitation Association Recommended Framework for Cardiac Rehabilitation 04* [14]. Table 2 documents the topics covered in the questionnaire. The semi-structured interview guide was developed, tested and modified prior to use and included questions specific to Indigenous people by exploring the use of culturally appropriate approaches for Indigenous patients, demographic data, staff cultural awareness training, and the availability of Indigenous staff (Table 3). Quantitative data collection was based on CR coordinator reports at interview and the interviewer recorded this on the interview guide (Table 2). CR coordinators were asked to send a summary of data through via email if possible. Quantitative data was entered and analysed in SPSS.

**Table 2:** Semi-structured interview questionnaire

Component	Content
Core component of CR	*Inpatient CR:* Type of program; type of facility; main elements of inpatient CR (basic information and reassurance, supportive counselling, mobilization and resumption of activities, discharge planning, and referral to outpatient CR and ongoing care). *Outpatient CR:* Pre-cardiovascular surgery review; assessment, review and follow-up; low or moderate intensity physical activity; education, discussion and counselling; ongoing assessment and management, and monitoring and evaluation.
Program information	Type of health service; type of program delivery; patient eligibility; program duration and frequency; attendance fee; patients from diverse populations; and patient monitoring and referral for further medical review.
Referral and attendance	Patient referral pathway; patient attendance and completion numbers; demographic data; patient outcomes; and exit strategies for patients on completion.
Program coordination and multidisciplinary team	Dedicated program coordinator; multidisciplinary team availability; use of CR guidelines; sustainability of program and program and service contact details.

Key: *CR* Cardiac Rehabilitation

**Table 3:** Questions relating to Indigenous Patients

	Question
Culturally appropriate programs or approaches	Describe any programs or approaches that are used to meet the needs of diverse population groups?
	Describe if and how your program: a. Uses case management for Aboriginal and Torres Strait Islander or other culturally diverse patients that covers the process of care from tests and/or procedures through to CR? b. Uses education materials on common cardiovascular conditions, tests, interventions, medications and CR designed for Aboriginal and Torres Strait Islander people? c. Employs or has access to an Aboriginal Health Worker to assist you and your Aboriginal and Torres Strait Islander or other culturally diverse patients? d. Operates a buddy and/or mentoring systems for patients whose family or carer is not available to take part in the decision-making process or accompany them to hospital?
Demographic data	What are the demographics of the patients who attend your program? % Male % Female % Indigenous Average age
CR guidelines One of several guidelines listed	Which CR guideline(s) guide your program? National Health and Medical Research Council. *Strengthening Cardiac Rehabilitation and Secondary Prevention for Aboriginal and Torres Strait Islander Peoples: A Guide fro Health Professionals.*
Staff cultural awareness training	Explain any cultural awareness training that staff in your program undertake?
Availability of Indigenous staff Both questions included Aboriginal Health Worker	Do you have a multidisciplinary team involved in your program? If yes, please indicate all relevant health professionals that are available to your program and if possible indicate hours per week. If your program does not have a multidisciplinary team, what health professionals does your program have access to?

Cardiac rehabilitation services, Aboriginal Medical Services and program coordinators were identified through the Heart Foundation (HF) *Directory of Western Australian Cardiac Rehabilitation and Secondary Prevention Services 2012.* Services identified through this sampling frame were contacted and coordinators given an explanation of the study and invited to participate. A Participant Information Sheet was emailed and a 60 min interview scheduled at the coordinator’s convenience. Coordinators returned a signed consent form and were re-consented verbally at the beginning of the interview for their responses to be digitally recorded. The Western Australian Country Health Service and University of Western Australia Human Ethics Committees approved the study.

### Data analysis

Quantitative data was analysed using IBM SPSS Statistics 22 and descriptive statistics utilised to report both continuous and categorical data. Data was exported to Excel and qualitative data relating to Indigenous people analysed manually by the principal investigator.

## Results

### Services and geographical locations

**Fig. 1:**
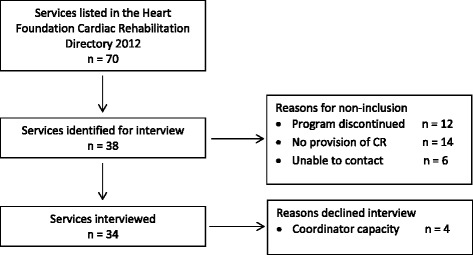
Flow chart of cardiac rehabilitation service identification and interview

Of the 70 services listed in the *Heart Foundation Cardiac Rehabilitation Directory 2012*, 38 were identified for interview as 12 programs were discontinued, 14 of 18 Aboriginal Medical Services (AMSs) reported not providing CR, and 6 services listed (including two AMSs) were not able to be contacted (Fig. 1). Two AMSs (one metropolitan and one rural) offered a specific chronic condition management program for their patients that included a strong focus on heart health. Thirty four programs (90 % of those identified for interview) were interviewed from March to July 2015; four declined due to a lack of capacity.

Of the programs identified for interview, 26 programs (68 %) were located in the four health regions in south west WA: the Wheatbelt, South West, Great Southern, and Metropolitan Perth. The remaining 32 % were spread across the Kimberley (*n* = 3), Pilbara (*n* = 3), Midwest (*n* = 4), and Goldfields (*n* = 2). The majority of services (68 %) were public hospital-based, 29 % were community-based and one private service was both hospital and community-based. Rural and remote services were mostly public hospital-based (93 %) compared with 42 % of metropolitan services. Metropolitan services were more likely to provide comprehensive CR programs (58 %) compared with rural (30 %) and remote services (17 %). Three regional programs offered dedicated CR exercise programs with *ad hoc* education and smaller rural and remote sites offered generic chronic condition exercise programs which cardiac patients could attend for CR. One remote Nursing Post offered telephone education and support.

Dedicated program coordinators were more likely to be physiotherapists (67 %) or registered nurses (24 %) (Table 4); two programs employed exercise physiologists and one program employed a health promotion officer. All rural and 83 % of remote program coordinators were physiotherapists and the hours dedicated to the role of program coordinator indicated it was a part time role (Table 4), with the majority stating it was only one component of their overall role. Program coordinators were more likely to be female (75 %) than male and this was evenly distributed over rural, remote and metropolitan programs. The length of time that the coordinators had worked in their role was widely variable and the range was wide in all program settings (Table 4).

**Table 4:** Demography of dedicated Cardiac Rehabilitation Coordinators

	Overall % (n)	Rural % (n)	Remote % (n)	Metropolitan % (n)
Health discipline of Coordinator				
• RN	18 (6)		17 (2)	34 (4)
• RN certified in CR	3 (1)			8 (1)
• Nurse Practitioner	3 (1)			8 (1)
• Physiotherapist	67 (23)	100 (10)	83 (10)	25 (3)
• Exercise Physiologist	6 (2)			17 (2)
• Health Promotion officer	3 (1)			8 (1)
Full time equivalent in CR role				
• ≤0.8 (range 0.2-0.8)	79 (27)	100 (10)	100 (12)	42 (5)
• 1.0	21 (7)	0 (0)	0 (0)	58 (7)
Months in position	Months	Months	Months	Months
• mean ± SD	52.8 ± 67.7	65.1 ± 97.2	48.5 ± 58.7	47.8 ± 53.4
• Range (min-max)	1.0 - 276	3.0 - 276	1.0 - 192	3.0 – 192

Key: *RN* Registered nurse, *CR* Cardiac rehabilitation

### Identification of and services for Indigenous patients

Overall, 65 % of coordinators reported that there were processes for identification of Indigenous Australians in their service. A higher proportion of services (75 %) in remote areas reported identification of Indigenous people compared with 50 % in rural and 67 % in metropolitan settings (Table 5). Western Australia (and Australia) has no standard system of CR data collection so our quantitative analysis is limited, based on coordinator reports rather than analysis of consistent, routinely collected service data. All program coordinators reported receiving patient referrals from metropolitan public hospitals. Other referral sources included regional public hospitals (50 %), metropolitan (60 %) and regional private hospitals (35 %), general practice (60 %), Aboriginal Medical Services (50 %) and self-referrals (40 %). One coordinator received referrals from private cardiology rooms and two coordinators situated in a remote service received referrals from remote Aboriginal community clinics. Cardiac rehabilitation coordinators, nursing and allied health staff and cardiologists were most likely to make the referral and the most common method of referral was a referral letter or a copy of the discharge letter. Other methods of referral included telephone calls, facsimile, internal eReferral and email. The length of time from discharge to receipt of referral varied from on discharge (46 %), one to two weeks (15 %), two to four weeks (18 %), one to three months (12 %) and other (9 %).

**Table 5:** Identifying and meeting the needs of Indigenous Australians

	Overall % (n)	Rural % (n)	Remote % (n)	Metropolitan % (n)
Identification of Indigenous Australians - % Yes	65 (34)	50 (10)	75 (12)	67 (12)
Case Management for Indigenous Australians - % Yes	32 (34)	50 (10)	17 (12)	33 (12)
Specific Educational Materials for Indigenous Australians - % Yes	35 (34)	20 (10)	25 (12)	58 (12)
Access to AHW – % Yes	71 (34)	80 (10)	83 (12)	50 (12)
Buddy or Mentoring System for Indigenous Australians - % Yes	27 (34)	30 (10)	25 (12)	25 (12)
Cultural awareness CPD for staff – % Yes	97 (33)	100 (10)	100 (12)	91 (11)
CR guidelines – % Yes	77 (33)	60 (10)	67 (12)	100 (11)

Reported referral rates varied widely and were greater for metropolitan services than for rural and remote services. The majority of rural and remote services received less than 5 Indigenous and non-Indigenous cardiac referrals per month, some less than 5 per year. The percentage of patients attending CR programs who are Indigenous varied widely across the regions: 50-75 % in the Kimberley, 5-25 % in the Pilbara, and 0-10 % in other regional areas. Metropolitan services reported between zero and less than 5 %; many respondents suggesting that Indigenous patients were referred to the AMS. Both AMS-based programs reported that 100 % of their participants were Indigenous. There was a disparity between genders attending CR as two thirds of patients (Indigenous and non-indigenous) who attended were male (63 % male vs 37 % female overall, 60 % vs 40 % rural and metropolitan and 66 % vs 33 % at remote services). Attendance rates for Indigenous patients was collected but not by gender. Available data indicates a wide range of program completion of patients who commenced CR: a range of 30-100 % overall with CR completion of 30-100 % in rural, 10-90 % in remote and 65-90 % in metropolitan services.

For the majority of programs (88 %) there was no participation fee. Two remote programs charged minimum fees for either the Heart Moves program (gold coin donation) or the first class at a Recreation Centre ($3.50). Two private metropolitan programs charged a fee with the amount of reimbursement of out-of-pocket expenses dependent on the private health insurance fund. Transport was the other cost for patients and related to private car use and parking or public transport use. Public transport was more accessible in metropolitan settings. Many programs had some measures in place to assist patients with transport: these included an AMS or voluntary bus service, taxi vouchers, Home and Community Care Program (HACC) services, and Aboriginal Liaison Officers providing transport.

### Programs to meet cultural needs of Indigenous patients

Overall, a third or less of the CR services provided specific programs or activities to meet the cultural needs of Indigenous patients (Table 5). Case management for Indigenous patients was offered by 32 % of CR services: this was higher for rural programs (50 %) compared with remote (17 %) and metropolitan (33 %). Specific educational materials for Indigenous patients were provided by 35 % of CR services: this was greater for metropolitan programs (58 %) compared with rural and remote programs (20 % and 25 % respectively). A buddy or mentoring system for Indigenous patients was offered by 27 % of programs with a similar proportion by rurality. Overall 71 % of programs reported being able to access an Aboriginal Health Worker with rural and remote programs (80 % and 83 % respectively) having greater access than metropolitan programs (50 %).

Metropolitan services reported that they referred Indigenous patients to the Indigenous specific program provided by the AMS for CR and secondary prevention. In the regional area with an AMS chronic condition program, it was reported that most Indigenous patients accessed the AMS program. Remote services were more likely to identify Indigenous people (Table 5) and provide additional activities to meet the cultural needs of Indigenous patients (83 %) compared with rural (30 %) and metropolitan (25 %) services, particularly in those regions in northern WA with a higher proportion of Indigenous people (100 %). Culturally appropriate activities included separate classes for men and women, home visits, visits to Aboriginal Communities, an Indigenous Therapy/Allied Health Assistant leading the classes, and a specific Indigenous group with a breakfast club and exercise class.

### Cultural awareness professional development

Overall, 97 % of coordinators (n = 33) reported receiving cultural awareness training within their organisation, 100 % of rural and remote and 91 % (n = 11) of metropolitan programs (Table 5). One metropolitan program did not receive cultural awareness training and a response was not recorded from another. A range of cultural awareness training options were utilised. In rural and remote regions training was most commonly provided through the mandatory Western Australian Country Health Services (WACHS) online training which in some instances was supplemented by video conference training, or face-to-face training from a local Aboriginal Corporation or local Language and Cultural Centre. Non-WACHS programs utilised a Heart Foundation presentation on Aboriginal CR or a university cultural awareness program. Cultural awareness delivery in the metropolitan setting included hospital-based e-learning supplemented by optional Indigenous cultural study days. Community programs included training from the Heart Foundation, Aboriginal Health Council of WA, Aboriginal Medical Service and the Health Department when staff also worked clinically in a hospital setting.

### Cardiac rehabilitation guideline use

Overall, 77 % of coordinators reported that their program was based on CR guidelines: 60 % rural, 67 % remote, and 100 % of metropolitan programs (Table 5). The National Heart Foundation of Australia and Australian Cardiac Rehabilitation Association *Recommended Framework for Cardiac Rehabilitation’04* [14] was the most frequently utilised (60 %) CR guideline. The ACRA *Core Components of Cardiovascular Disease Secondary Prevention and Cardiac Rehabilitation 2014* [43] and the 2014 Department of Health, Western Australia, *Cardiovascular rehabilitation and secondary prevention pathway principles for Western Australia* [44] were utilised by 15 % of the CR services. No coordinators reported utilising the National Health and Medical Research Council, *Strengthening Cardiac Rehabilitation and Secondary Prevention for Aboriginal and Torres Strait Islander Peoples: A Guide for Health Professionals* [41]. Twenty percent of programs utilised more than one set of guidelines, including WA Models of Care and interstate or international programs/ guidelines such as the Flinders Chronic Condition Management Program [45, 46], Statewide Cardiology Clinical Network’s *Cardiac Rehabilitation: a Model of Care for South Australia* [47] or American Heart Association’s *Cardiac Rehabilitation and Secondary prevention of Coronary Heart Disease Scientific Statement* [18]. A minority (9 %) of CR Coordinators were unsure of the guidelines utilised and 6 % stated that their program was “not really” guideline based.

## Discussion

Multifaceted strategies to promote access to flexible CR and secondary prevention services tailored to meet the needs, preferences and cultural safety of individuals are required [10]. Patient-centred access to health care is complex and as conceptualised by Levesque et al., includes 5 dimensions: Approachability; Acceptability; Availability; Affordability; and Appropriateness [48]. According to Levesque there are 5 corresponding abilities needed to interact with the dimensions of accessibility: Ability to perceive; Ability to seek; Ability to reach; Ability to pay; and Ability to engage [48]. The *Strengthening Cardiac Rehabilitation and Secondary Prevention for Aboriginal and Torres Strait Islander Peoples: A Guide for Health Professionals* was developed to provide strategies for improved access to and uptake of CR for Indigenous Australians. [41]. In this study, despite evidence of much good practice, we demonstrate a continuing access gap to CR and secondary prevention services for Indigenous Australians in WA.

### Access to CR services in Western Australia

Lack of referral to and enrolment in CR has been significantly related to a drive time of over 60 min so geographical location is a major determinant of access to CR for many people living rurally [29]. Clark et al. reported that approximately 95 % of Australians live within 1 h of basic services to support CR and secondary prevention, including 75 % of the Indigenous population [49]. Conversely, they report that 14 % of Indigenous people live in geographical regions with poor access to health services that provide CR [49]. In WA, 63.2 % of the Indigenous population live outside of Metropolitan Perth with higher proportions living in the Kimberley (43 %), Pilbara (16 %), Midwest (12 %), and Goldfields (12 %). However, only 31.6 % of CR services were located in larger regional cities or towns of the Kimberley, Pilbara, Midwest and Goldfields, an area of some 2,312,237 km^2^ or 87.4 % of the total WA land mass. Thus a major access barrier for many Indigenous patients in WA is the lack of available CR service provision and the challenge of reaching services. Alternative methods of CR delivery such as home-based CR via telehealth or mobile health platforms may overcome this geographical isolation [11].

The *Strengthening Cardiac Rehabilitation and Secondary Prevention for Aboriginal and Torres Strait Islander Peoples: A Guide for Health Professionals* recommend that CR for Indigenous patients draw on existing CR and secondary prevention services as appropriate (Table 1, key Measure 3) [41]. We identified a limited number of services that provided CR and secondary prevention specific to Indigenous patients. Given that CR programs were not generally provided in AMS’s in WA, it is essential that CR services ensure cultural safety is integral to their core business and they take the specific needs of Indigenous Australians into consideration in planning and delivering CR, including alternative methods of CR delivery (Table 1, Key measures 1 and 6).

### Identification, participation and completion rates for Indigenous patients

To effectively improve access to CR and meet the cultural needs of Indigenous people, they must firstly be identified as Indigenous. Thompson et al., reported that fewer than half (47.7 %) of CR services had processes in place for identifying Indigenous status [40]. Our findings demonstrate improvement in this, especially in remote WA, but there are opportunities to improve this in one third of services not currently identifying Indigenous status.

Shepherd and colleagues reported low Indigenous participation in structured general practice-based CR in remote Queensland [38]. Lack of knowledge about CR, possibly relating to a lack of referral was demonstrated as a barrier to CR participation [38]. To overcome such disparities requires coordination to enable continuity of care for Indigenous patients. Integrated pathways are likely to occur when there are effective partnerships and information flow between health services and referral networks [41, 42]. Metropolitan public hospitals were identified by all CR coordinators as a referral pathway but referrals from other health care providers such as private hospitals or general practice was reported by less than two thirds of coordinators and made a minor contribution to referrals. This indicates a need for more effective information flow from all systems of care provision that a patient encounters following an acute cardiac event. Integrated clinical referral pathways, education and awareness raising of all health providers and electronic record alerts in general practice provide potential solutions to increase across system checks and referrals.

Coordinator responses indicate a wide variation in the number of Indigenous patients attending CR in WA, largely related to geographical location and population density. Respondents reported low numbers of referrals but a higher proportion of Indigenous attendance in northern WA, corresponding to a larger Indigenous population in these regions. A lower level of attendance at metropolitan services was attributed to referral of Indigenous patients to the AMS program although there is no data on the proportion of Indigenous patients who require CR who actually attend this program. The Perth metropolitan area covers 6,418 km^2^ so issues of distance still exist for Indigenous people who are more likely to live in the outer metropolitan suburbs, although the AMS’s provision of a transport service for patients is an important component of access.

Respondents had difficulty reporting attendance and completion rates for Indigenous and non-Indigenous patients for many reasons: the open-ended nature of some programs; the lack of a clear definition of completion; and inconsistencies in collection of data. A standardised and consistent method of data collection in Australia would enable accurate evaluation and could prompt advocacy for service improvement for Indigenous patients [22, 43]. The ACRA *Core Components of Cardiovascular Disease Secondary Prevention and Cardiac Rehabilitation 2014* [43] and the *Cardiovascular Rehabilitation and Secondary Prevention Pathway Principles for Western Australian* [44] recommend the collection of a minimum dataset to record and monitor CR practice in Australia and WA. The ACRA Core Components [43] offer a comprehensive list of data that may be collected, but for rural and remote services with limited capacity a minimum data set would be preferable.

### Programs to meet cultural needs of Indigenous patients

According to Levesque, for services to be accessible they must be acceptable and appropriate, thus allowing people to seek and engage [48]. For CR to be acceptable and appropriate it needs to take the personal, social and cultural needs of the patient into account and provide culturally appropriate care, resources and tools to empower self-management and autonomy (Table 1, Key measures 2, 4 and 6) [41, 48, 50]. Measures that met the cultural needs of Indigenous patients include the availability of an Aboriginal Health Professional [22, 41], availability of specific educational materials for Indigenous Australians [41], case-management by cardiac coordinators [42], and having a buddying or mentoring system in place [37], however, these are not routinely available [44]. In WA, the majority of CR services were able to access an Aboriginal Health Worker and/or Liaison Officer but a limited number of services were able to offer specific educational materials, case-management or a buddying system. Provision of alternative methods of home-based CR for Indigenous patients that incorporate a family and a community buddy [37, 42] with professional support from a cardiac coordinator and Indigenous Health Professional may help overcome the cultural barriers characteristic of traditional-CR services.

### Cultural awareness professional development

Cultural safety, integral to the core business of an organisation (Table 1, Key measure 1), requires specialist resource development and training for continuing professional development for all health professionals working in heart health care, including Aboriginal Health Workers, Liaison Officers and Allied Health Assistants (Table 1, Key measure 7) [41]. It is a health professional’s responsibility to ensure they receive cultural awareness training and practice in a culturally safe manner and the responsibility of the organisation to provide support and training to ensure that services are provided in a culturally respectful way [41]. The extent to which this form of training translates into the provision of culturally safe care is not clear, and it is important to realise that it is the whole system of care which will influence a patient’s experience of a service.

### Cardiac rehabilitation guideline use

Evidence-based guidelines are important in promoting best-practice and driving practice change [40]. This also applies to the promotion of cultural awareness and culturally safe practice. Organisations providing a CR service need to develop or utilise supportive protocols, policies and procedures that address the personal, social and cultural needs of patients (Table 1, Key measure 6) [41]. The *Strengthening Cardiac Rehabilitation and Secondary Prevention for Aboriginal and Torres Strait Islander Peoples: A Guide for Health Professionals in 2008* [40, 41] was an excellent resource that includes strategies and tools for the provision of culturally appropriate CR programs to improve the uptake and access to CR for Indigenous people. However, Thompson et al. reported suboptimal awareness and limited implementation in WA in 2008 and disturbingly no CR service was utilizing this guide in 2015.

The ACRA *Core Components of Cardiovascular Disease Secondary Prevention and Cardiac Rehabilitation 2014* [43] and the *Cardiovascular rehabilitation and secondary prevention pathway principles for Western Australia* [44] acknowledge the barriers to Indigenous patients accessing CR and the need for patient-centred culturally appropriate CR services. Details of culturally appropriate patient resources were provided but they did not discuss specific strategies or resources for providing culturally appropriate care nor did they reference the *Strengthening Cardiac Rehabilitation and Secondary Prevention for Aboriginal and Torres Strait Islander Peoples: A Guide for Health Professionals* [41] which if utilised would help guide health professionals working in CR services to provide better care for Indigenous patients.

### Strengths, limitations and implications

The strength of our study relates to the in-depth assessment undertaken, the rich level of data obtained from the CR coordinators and high participation of services in the study. Limitations arise from CR services in WA and Australia lacking a standard method of data collection which limits the availability of quantitative client data and our ability to make direct comparisons between services and report inferential statistics. All three guidelines previously discussed recommend the collection of information to identify problem areas and measure change [41], a minimum dataset to record and monitor CR and secondary prevention in WA [44] that could be used to evaluate the quality and outcome of an individual’s participation for each of the five core components of CR [43]. A further limitation is the inability to estimate the number of Indigenous patients who are not referred to CR, as access to concurrent discharge data for the number of eligible patients was not included in the study design. The relatively high level of completion of CR described by coordinators indicates that an essential step in CR delivery is referral and initial attendance. Finally, there is an absence of Aboriginal people’s views of CR service provision in this study, a gap that has been previously identified [37, 51, 52] but there are relatively few reports in the literature.

Our results have important implications for CR and secondary prevention delivery for Indigenous patients in Western Australia. Ongoing quality improvement of CR services and the provision of culturally appropriate, flexible, accessible and guideline-based alternative methods of CR delivery need to be explored. Continued efforts to ensure Indigenous status is identified, endorsement of guideline implementation, and a structured integrated health pathway to ensure coordinated management of CR and secondary prevention are essential. Awareness raising and continuing professional development for all health professionals involved in supporting the patient’s journey is necessary to improve knowledge of and use of CR guidelines. Our findings suggest that a minimum data set enabling assessment of service key performance indicators for all phases of CR could underpin quality improvement and improve tracking of patient referral, attendance, adherence and completion rates. If implemented at a national level, there are opportunities to establish relevant benchmarks and accountability for service function in urban, rural and remote settings.

Advances in technology provide avenues to add value to centre-based CR services. Mobile health technology has been successfully trialled for the delivery of a comprehensive CR program in Brisbane, with improved uptake and completion rates and improved health outcomes equal to centre-based CR [53]. Application of this technology has great potential for improving access in rural and remote Australia but is still to be tested in these settings and its utility to support Indigenous people remains to be assessed.

### Future directions

To address the findings from this in-depth assessment of CR services in WA, the authors and partners are progressing the implementation and evaluation of an innovative and validated mobile health platform for the delivery of home-based comprehensive CR in WA. A context-sensitive implementation study will utilise a collaborative participatory approach with the Indigenous community and health service providers to ensure the program is culturally appropriate for Indigenous and non-Indigenous Western Australians.

## Conclusions

The under-representation of Indigenous Australians participating in CR, as reported in the literature and more specifically in this study, mandates a concerted effort to improve services to better meet the needs of Indigenous patients with CVD as part of closing the gap in life expectancy. Improving access to culturally appropriate CR and secondary prevention in Western Australia must be an important component of this effort given the high rates of premature cardiovascular disease affecting Indigenous people. Our findings highlight the importance of good systematic data collection across services, establishing benchmarks that enable regular monitoring and upskilling of all health staff involved in supporting CVD patients to ensure they are aware of CR guidelines and promote uptake. Health pathways that ensure continuity of care and alternative methods of CR delivery with dedicated resources are needed.

## Abbreviations

ACRA, Australian Cardiovascular Health and Rehabilitation; AHW, Aboriginal Health Worker; AMS, Aboriginal Medical Service; CHD, Coronary Heart Disease; CPD, Continuing Professional Development; CR, Cardiac rehabilitation; CVD, Cardiovascular disease; HACC, Home and Community Care Program; NHMRC, National Health and Medical Research Council; WA, Western Australia; WACHS, Western Australian Country Health Service
